# Supplementary material for: Variation of Glucosinolate Contents in Clubroot-Resistant and -Susceptible Brassica napus Cultivars in Response to Virulence of Plasmodiophora brassicae
Source: Pathogens. 2021 May 6;10(5):563. doi: 10.3390/pathogens10050563 (PMC8148440; doi:10.3390/pathogens10050563)
Supplement: Supplementary file 1 [file pathogens-10-00563-s001.zip › pathogens-1163969-supplementary.pdf]

Supplementary Table S1: *P*-values of mean glucosinolate contents in leaves analyzed with Tukey`s post hoc tests for the means of the factor 'pathogen'. *P*-values show the significance of differences in glucosinolate contents between the single resistant varieties (Aristoteles, Creed, Mendel) and the single susceptible varieties (Bender, Ladoga, Visby) averaged over the factor 'pathogen' (control, P1, P1 (+)). Supplemental information for Figure 1.

	**Aristoteles – Bender**	**Aristoteles - Ladoga**	**Aristoteles – Visby**	**Creed – Bender**	**Creed - Ladoga**	**Creed – Visby**	**Mendel – Bender**	**Mendel - Ladoga**	**Mendel – Visby**
Progoitrin	0.79	0.97	0.99	0.35	0.23	0.086	0.10	0.65	0.26
Glucobrassicin	0.57	0.40	0.51	0.82	0.62	0.76	1.0	0.99	1.0
4-Methoxyglucobrassicin	0.93	0.70	0.80	0.83	0.55	0.66	0.99	0.56	0.32
Neoglucobrassicin	0.89	1.0	0.38	0.92	1.0	0.41	0.93	1.0	0.42

Supplementary Table S2: *P*-values of mean glucosinolate contents in leaves analyzed with Tukey`s post hoc tests for the means of the factor 'variety'. *P*-values show the significance of differences in glucosinolate contents between the mean of the resistant varieties (Aristoteles, Creed, Mendel) and the mean of the susceptible varieties (Bender, Ladoga, Visby) averaged over the factor 'variety' (resistant, susceptible). Experiment: differences in glucosinolate contents between two independent Experiments; *P. brassicae*-P1: differences in glucosinolate contents between resistant and susceptible cultivars inoculated with P1; *P. brassicae*-P1 (+): differences in glucosinolate contents between resistant and susceptible cultivars inoculated with P1(+); Trait: differences in glucosinolate contents between resistant and susceptible cultivars averaged over the two pathotypes (P1 and P1 (+). Supplemental information for Figure 2.

	**Experiment**	***P. brassicae*-P1**	***P. brassicae*-P1 (+)**	**Trait**
Progoitrin	0.48	0.012	0.0055	0.013
Glucobrassicin	0.73	0.026	0.30	0.096
4-Methoxyglucobrassicin	0.031	0.019	0.018	0.038
Neoglucobrassicin	0.83	0.52	0.028	0.14
Aliphatic glucosinolates	0.70	0.005	0.0024	0.0071
Indolic glucosinolates	0.31	0.0031	0.011	0.0124

Supplementary Table S3: *P*-values of mean glucosinolate contents in roots analyzed with Tukey`s post hoc tests for the means of the factor 'variety'. *P*-values show the significance of differences in glucosinolate contents between the mean of the resistant varieties (Aristoteles, Creed, Mendel) and the mean of the susceptible varieties (Bender, Ladoga, Visby) averaged over the factor 'variety' (resistant, susceptible). Experiment: differences in glucosinolate contents between two independent Experiments; *P. brassicae*-P1: differences in glucosinolate contents between resistant and susceptible cultivars inoculated with P1; *P. brassicae*-P1 (+): differences in glucosinolate contents between resistant and susceptible

cultivars inoculated with P1(+); Trait: differences in glucosinolate contents between resistant and susceptible cultivars averaged over the two pathotypes (P1 and P1 (+). Supplemental information for Figure 3.

	**Experiment**	***P. brassicae*-P1**	***P. brassicae*-P1 (+)**	**Trait**
Progoitrin	0.14	0.19	0.17	0.20
Glucoalyssin	0.34	0.46	0.37	0.32
Glucobrassicin	0.44	0.20	0.20	0.15
4-Methoxyglucobrassicin	0.15	0.83	0.19	0.89
Gluconasturtiin	0.81	0.067	0.56	0.28
Neoglucobrassicin	0.65	0.14	0.38	0.15
